# Incidence and risk factors for recurrence of incisional hernia repair after liver transplantation: a retrospective cohort study

**DOI:** 10.1007/s10029-025-03400-0

**Published:** 2025-06-27

**Authors:** Edouard Wasielewski, Thierry Pecot, Estelle Le Pabic, Mohamed Lakehal, Astrid Herrero, Fabien Robin, Karim Boudjema, Laurent Sulpice

**Affiliations:** 1 Department of Hepatobiliary and Digestive Surgery, University Hospital, Rennes 1 University, Rennes, France; 2 University of Rennes, Biosit UAR 3480 CNRS– US 018 Inserm, FAIIA core facility, Rennes, F-35000 France; 3 Department of Clinical PharmacologyUniversity Hospital, Rennes 1 University, Rennes, France; 4 Department of HBP Surgery & Transplantation, St-Eloi University Hospital, Montpellier, France; 5 Department of Digestive Surgery, CHU Rennes 35 000, Pontchaillou Hospital, Rennes 1 University, Rennes, France

**Keywords:** Liver transplantation, Recurrence, Incisional hernia, Skeletal muscle index

## Abstract

**Purpose:**

The objective of this study was to evaluate the incidence and identify risk factors associated with the recurrence of incisional hernia (IH) following initial parietal repair in patients who had undergone liver transplantation. Liver transplantation (LT) is a complex procedure associated with numerous complications, particularly those affecting the abdominal wall, with incisional hernia (IH) being the most significant. To date, the risk factors contributing to IH recurrence in this population have not been systematically assessed.

**Methods:**

This retrospective, single-center study included all patients who underwent liver transplantation at Rennes University Hospital between January 1, 2012, and December 31, 2018. The list of eligible patients was provided by the French Biomedicine Agency.

**Results:**

A total of 803 liver transplant recipients were included. Among them, 282 patients (35.1%) developed an incisional hernia (IH) after a median follow-up of 75.87 months [54.7–97.2]. Of these, 50 patients (35.4%) experienced IH recurrence at a median interval of 8.8 months [5.2–34.2] following primary repair. Identified risk factors for recurrence included: length of hospital stay after the initial repair (HR: 1.09 [1.09–1.13], P = < 0.0001), type of repair (suture versus prosthetic) (HR: 2.48 [1.25–4.9], P = 0.009). Visceral obesity is considered a high-risk factor (HR: 2.21 [0.98–4.95], P = 0.055), although the association no longer reached the conventional threshold for statistical significance.”

**Conclusion:**

To the best of our knowledge, this is the first study to assess body composition as a risk factor for incisional hernia recurrence in the liver transplant population. These findings highlight the importance of considering visceral obesity as a significant predictor of recurrence.

**Supplementary Information:**

The online version contains supplementary material available at 10.1007/s10029-025-03400-0.

## Introduction

Liver transplantation (LT) remains a critical intervention for patients with advanced liver disease. Despite significant medical and surgical advances, postoperative complications are frequent, among which incisional hernias (IH) are notably prevalent. IH, defined as a defect in the abdominal wall at the site of a previous surgical incision, affects up to 35.6% of the general population [1], with reported incidence rates ranging from 1.7 to 43% in liver transplant recipients [2]. In addition to well-established risk factors [3–5], several other variables—including advanced age [6], a history of pulmonary disease [2], incision type [7], diabetes mellitus [8], and immunosuppressive therapy [9]—have been implicated in the development of IH within this specific patient population.

Assessment of nutritional status, particularly body composition, is of critical importance in the management of patients both before and after liver transplantation [10]. Sarcopenia—defined by the European Working Group on Sarcopenia in Older People (EWGSOP) as a loss of muscle mass associated with adverse outcomes such as falls, fractures, and increased mortality [11]—is highly prevalent among individuals with cirrhosis [12] and tends to worsen with disease progression [13]. Moreover, obesity, frequently coexisting with sarcopenia in the form of sarcopenic obesity, has been identified as a prognostic factor for post-transplant mortality [14].

Body composition can be assessed using various techniques, including dual-energy X-ray absorptiometry (DEXA) and computed tomography (CT). Although DEXA is considered the gold standard, CT scanning offers a faster and more widely accessible means of evaluating both sarcopenia and visceral obesity in patients awaiting liver transplantation [15].

The aim of the present study was to identify prognostic factors associated with the recurrence of incisional hernia following liver transplantation. We hypothesized that both sarcopenia and visceral obesity contribute to an increased risk of recurrence.

## Patients and methods

### Study design

We conducted a retrospective analysis of all adult patients who underwent liver transplantation at Rennes University Hospital between January 1, 2012, and December 31, 2018. The list of transplant recipients was obtained from the French Biomedicine Agency. All surviving patients were informed of the study and provided their consent to participate. The study was conducted in accordance with the ethical principles outlined in the 2013 declarations of Helsinki and Istanbul, and received approval from the Ethics Committee of Rennes University Hospital (No. 25.21).

### Patients

Data prospectively collected by the Biomedicine Agency up to the time of liver transplantation included demographic and anthropometric characteristics (age, sex, weight, height, body mass index [BMI]), medical history, etiology of liver disease, and the severity of cirrhosis as assessed by both the Child–Pugh–Turcotte score and the Model for End-Stage Liver Disease (MELD) score. Additional data, retrospectively obtained from patients’ medical records, included smoking status, pre-transplant serum albumin levels, treatments received prior to transplantation (e.g., chemotherapy, radiotherapy, microwave ablation), and any history of liver surgery (including hepatectomy and atypical resections) prior to transplantation.

### Incisional hernia or recurrence

The diagnosis of incisional hernia (IH) or its recurrence was made clinically and confirmed by contrast-enhanced computed tomography (CT) in cases of diagnostic uncertainty, particularly in obese patients. Indications for IH repair included aesthetic discomfort, pain, or impaired ability to resume physical activity. No emergency surgeries were performed for hernia strangulation. Collected data included the date of onset, anatomical location, type of surgical repair, and postoperative complications occurring within 30 days. Complications were classified according to the Clavien-Dindo system, with severe complications defined as grade > IIIa [16]. Surgical repair consisted of either simple suture or mesh placement, depending on the size of the hernia and the local risk of infection.

## Evaluation of muscle and adipose mass

### Automation of measurement

Evaluation of muscle and adipose tissue mass was conducted using the most recent computed tomography (CT) scan performed within 90 days prior to liver transplantation. Analysis was based on a single axial slice at the level of the third lumbar vertebra (L3). Tissue differentiation was performed using Hounsfield unit (HU) thresholds: − 29 to + 150 HU for skeletal muscle [17], and − 190 to − 30 HU for adipose tissue [15]. The skeletal muscle area, normalized to the square of the patient’s height, was used to calculate the Skeletal Muscle Index (SMI). Thresholds defining sarcopenia were set at < 38.5 cm²/m² for females and < 52.4 cm²/m² for males [18]. Visceral obesity, assessed via Visceral Fat Area (VFA), was defined by a threshold of > 100 cm² for both sexes [19]. Subcutaneous fat was measured as Subcutaneous Fat Area (SFA), expressed in cm²; in the absence of established cut-off values, raw data were used for analysis.

The segmentation protocol and automation process for measurement have been previously described [20]. The complete software and accompanying video tutorials are available at: https://github.com/tpecot/MuViSS.

### Statistical analysis

In this study, quantitative variables are presented as mean ± standard deviation or as median with interquartile ranges (Q1–Q3), while qualitative variables are expressed as percentages. Time to first incisional hernia (IH) was calculated from the date of liver transplantation, and time to recurrence from the date of initial hernia repair. Patients who did not have a recurrence during the study period were considered as censored at the time of their last follow-up visit. Recurrence-free survival was defined as the interval between the date of the first IH repair and the date of recurrence, and was estimated using the Kaplan–Meier method. Variables potentially associated with recurrence were first assessed through univariate analysis. Variables with a significance level of *P* < 0.20 were included in a Fine and Gray subdistribution hazards model to appropriately account for competing risks. Stepwise backward selection was then applied to identify independent predictors of recurrence. Statistical significance was set at *P* < 0.05. All statistical analyses were performed using SAS software, version 9.4^®^ (SAS Institute Inc., Cary, NC, USA).

## Results

Between January 1, 2012, and December 31, 2018, 804 patients underwent LT at the University Hospital of Rennes, and 803 were selected for this study. One transplant patient who remained in the department for more than two years for administrative reasons was excluded given that he was not subject to the same risks as transplant patients who returned to life outside the hospital. Of the 803 transplanted patients and after a median follow-up of 75.87 months (IQR 54.7–97.2), 35.1% (*n* = 282) developed a first IH. The median time of occurrence of this first IH was 10.2 months (IQR 5.0–23.6). Of the 282 transplant recipients who developed IH, 50% (*n* = 141) underwent open surgical repair. The median time from diagnosis to repair was 10.8 months (IQR 4.6–15.3). Of the 141 graft recipients who received open surgical repair of the IH, 35.4% (*n* = 50) presented recurrence. The median time from repair of the first IH to recurrence was 8.8 months ((IQR 5.2–34.2). Of the 50 transplant recipients who redeveloped a recurrence, 54% (*n* = 28) had a redo repair. The median time from recurrence to repair was 5.33 months (IQR 3.0–8.0). Table 1 reports the characteristics of the patients in the study population according to whether or not they developed recurrence. Only the BMI (28.3 (IQR 25.9–32.3) vs. 26.26 (IQR 23.9–29.3) (P value = 0.006) was significantly higher in the group of patients who presented recurrence. There was no other significant difference between the 2 groups. The cumulative incidence without IH and the cumulative incidence without recurrence as a function of time are shown in the supplementary data (Fig. S1).

**Table 1:** Characteristics of patients treated for a first IH

		Recurrence			
Variable	Global	No	Yes	HR [IC95%]	*P*-value
**Age**,** median (years)**	60. 0 [51.0–64.0]	60.0 [51.0–64.0]	60.5 [51.0–63.0]	0.99 (0.9; 1.0)	0.59
**Sex**,** N (%)**					0.13
Female	16 (11.3%)	13 (14.3%)	3 (6.0%)	1	
Male	125 (88.7%)	78 (85.7%)	47 (94.0%)	2.47 (0.8; 7.9)	
**BMI**,** median (kg/m**^**2**^**)**	27.1 [24.6–30.5]	26.26 [23.9–29.3]	28.29 [25.9–32.3]	1.08 (1.0; 1.1)	0.006
**Tobacco use**,** N (%)**					0.21
No	36 (28.8%)	21 (25.3%)	15 (35.7%)	1	
Yes	89 (71.2%)	62 (74.7%)	27 (64.3%)	0.67 (0.4; 1.3)	
**Diabetes**,** N (%)**					0.94
No	105 (74.5%)	68 (74.7%)	37 (74.0%)	1	
Yes	36 (25.5%)	23 (25.3%)	13 (26.0%)	0.98 (0.5; 1.8)	
**Albumin before LT median (g/dL)**	33.8 [29.2–39.0]	32.9 [29.3–38.2]	36.0 [28.2–39.5]	1.01 (0.9; 1.1)	0.55
**Etiology**					0.90
Alcohol	44 (31.2%)	15 (30.0%)	29 (31.9%)	1	
Dysmetabolic cirrhosis	1 (0.7%)	0 (0.0%)	1 (1.1%)	0.7 (0.1; 12.9)	
Mixed cirrhosis	9 (6.4%)	3 (6.0%)	6 (6.6%)	0.96 (0.3; 3.2)	
Viral	6 (4.3%)	3 (6.0%)	3 (3.3%)	1.57 (0.5; 5.2)	
HCC	66 (46.8%)	25 (50.0%)	41 (45.1%)	1.09 (0.6; 2.1)	
Other	15 (10.6%)	4 (8.0%)	11 (12.1%)	0.66 (0.2; 2.0)	
**MELD**,** median**	17.0 [9.50–24.0]	17.0 [9.0–24.5]	17.0 [10.0–24.0]	1.00 (0.9; 1.0)	0.86
**Surgery before LT**					0.06
No	103 (73.0%)	32 (64.0%)	71 (78.0%)	1	
Yes	38 (27.0%)	18 (36.0%)	20 (22.0%)	1.72 (0.9; 3.1)	
**IS ≥ 2**,** N (%)**					0.70
Yes	118 (88.1%)	39 (86.7%)	79 (88.8%)	1	
No	16 (11.9%)	6 (13.3%)	10 (11.2%)	0.85 (0.4; 2.0)	

BMI: Body mass index– HCC: Hepatocellular Carcinoma– HR: Hazard Ratio - IS: immunosuppressors - LT: Liver transplantation– MELD: Model for End-Stage Liver Disease

The anatomic characteristics of the first IH are represented in Table 2. Recurrences were more frequent in transplant recipients whose first IH was repaired by running suture.

**Table 2:** Characteristics of first parietal repair

Variable	Global	Recurrence		HR [IC95%]	*P*-value
		No	Yes		
**Localisation**,** N (%)**					0.07
Double	12 (8.5%)	7 (7.7%)	5 (10.0%)	1	
Junction	12 (8.5%)	5 (5.5%)	7 (14.0%)	1.74 (0.6; 5.5)	
Transversal	33 (23.4%)	21 (23.1%)	12 (24.0%)	0.71 (0.3; 2.0)	
Vertical	84 (59.6%)	58 (63.7%)	26 (52.0%)	0.57 (0.2; 1.5)	
**Type of repair**,** N (%)**					0.02
Prothesis	101 (73.7%)	70 (80.5%)	31 (62.0%)	1	
Suture	36 (26.3%)	17 (19.5%)	19 (38.0%)	1.91 (1.1; 3.4)	
**Localisation of prothesis**,** N (%)**					0.51
Intraperitoneal	65 (64.4%)	41 (58.6%)	24 (77.4%)	1	
Retromuscular	36 (35.6%)	29 (41.4%)	7 (22.6%)	0.76 (0.3; 1.7)	

HR: Hazard Ratio

The rate and severity of complications that occurred after the first IH repair according to whether the grafters recurred or not are reported in Table 3. Postoperative complications as well as their severity did not influence recurrence. Recurrence of IH was significantly associated with the risk of removal of the mesh (6 (17.1%) vs. 2 (3.3%) (P value = 0.02) as well as with an increase in the median length of hospital stay (9.0 (IQR 7.0–15.0) vs. 7.5 (IQR 6.0–10.0) (P value < 0.0001).

**Table 3:** Post-operative complication

Variable	Global	Recurrence		HR [IC95%]	*P*-value
		No	Yes		
**Complication**,** N (%)**					0.17
No	82 (62.6%)	56 (67.5%)	26 (54.2%)	1	
Yes	49 (37.4%)	27 (32.5%)	22 (45.8%)	1.49 (0.8; 2.6)	
**Clavien-Dindo score**,** N (%)**					0.54
I	61 (48.8%)	42 (53.2%)	19 (41.3%)	1	
II	42 (33.6%)	26 (32.9%)	16 (34.8%)	1.39 (0.7; 2.7)	
IIIa	0 (0%)	0 (0.0%)	0 (0.0%)		
IIIb	17 (13.6%)	8 (10.1%)	9 (19.6%)	1.66 (0.8; 3.7)	
IVa	4 (3.2%)	2 (2.5%)	2 (4.3%)	1.80 (0.4; 7.8)	
IVb	0 (0.0%)	0 (0.0%)	0 (0.0%)		
V	1 (0.8%)	1 (1.3%)	0 (0.0%)		
**Re-intervention**,** N (%)**					0.32
No	97 (82,2%)	63 (85,1%)	34 (77,3%)	1	
Yes	21 (17,8%)	11 (14,9%)	10 (22,7%)	1.67 (0.6; 4.9)	
**Ablation of prothesis**,** N (%)**					0.01
No	87 (91.6%)	58 (96.7%)	29 (82.9%)	1	
Yes	8 (8.4%)	2 (3.3%)	6 (17.1%)	2.99 (1.2; 7.2)	
**Hospital stay**,** median (days)**	8.0 [6.0–12.0]	7.5 [6.0–10.0]	9.0 [7.0–15.0]	1.08 (1.0; 1.1)	< 0.001

HR: Hazard Ratio

Body composition represented by muscle mass and parietal or visceral fat mass according to whether IH recurred or not are reported in Table 4. In our study, the rate of sarcopenic patients was not statistically different between the 2 groups. The VFA (207.0 (IQR 118.0–284.0) vs. 145.0 (IQR 86.5–220.5)) (P value = 0.03) and the presence of visceral obesity (37 (84.1%) vs. 57 (69.5%)) (P value = 0.0384) were significantly higher in the group of patients with recurrence. Parietal fat mass, represented by SFA, was not significantly different between the 2 groups.

**Table 4:** Body composition of patients with an IH

Variable	Global	Recurrence		HR [IC95%]	*P*-value
		No	Yes		
**Visceral obesity**,** N (%)**					0.03
Yes	94 (74.6%)	57 (69.5%)	37 (84.1%)	1	
No	32 (25.4%)	25 (30.5%)	7 (15.9%)	2.36 (1.1; 5.3)	
**Sarcopenia**,** N (%)**					0.97
Yes	55 (43.7%)	36 (43.9%)	19 (43.2%)	1	
No	71 (56.3%)	46 (56.1%)	25 (56.8%)	1.01 (0.6; 1.8)	
**SFA**,** median (cm**^**2**^**)**	55.1 [36.6–85.91]	52.6 [34.1–85.9]	59.7 [38.9–88.8]	1.00 (0.9; 1.0)	0.55

HR: Hazard Ratio - SFA: Subcutaneous Fat Area - SMI: Skeletal Muscle Index - VFA: Visceral Fat area

The search for independent predictive factors for recurrence after a first cure is reported in Table 5. The length of hospital stay after surgical cure of the IH (HR: 1.09 (95% CI 1.06; 1.13)), running suture to repair a first IH (HR: 2.5 (95% CI 1.3; 4.9)). Although visceral obesity did not reach statistical significance in the multivariable model (HR: 2.21(95% CI: 0.98;4.95), the strength and consistency of the association suggest a strong trend toward increased risk.

**Table 5:** Independent prognostic factors for recurrence with the fine and Gray competing risks regression model

*N*	Variable	HR [95%CI]	*P*-value
113			.
.	Length of stay	1.09 [1.06; 1.13]	< 0.0001
.	Type of repair	2.48 [1.25; 4.90]	0.0091
.	Visceral obesity	2.21 [0.98; 4.95]	0.0553

HR: Hazard Ratio

## Discussion

Incisional hernia (IH) is a common and serious complication following liver transplantation (LT), often causing pain and discomfort during physical activity and carrying a potential risk of strangulation. Surgical repair is generally recommended, regardless of the technique or surgical approach employed.

In this retrospective, single-center study, we observed a high incidence of IH, affecting more than one-third of transplant recipients. Our findings are consistent with the existing literature, particularly the review conducted by *Garmpis et al.* [21]. It is noteworthy that only half of the initial hernias were repaired—primarily based on patient preference—and that the recurrence rate was comparable to the rate of initial occurrence. The repair rate observed in this study was slightly higher than that reported in the literature, which ranges from 39.4 to 43% [22, 23]. However, the recurrence rate observed in this study was slightly higher than that reported in the literature, which ranges from 3.4 to 25% [24, 25]. Several factors were found to be associated with a two- to three-fold increased risk of recurrence, including the use of simple suture repair and the presence of visceral obesity. Additionally, prolonged hospitalization following the initial repair further heightened this risk. Although sarcopenia is often regarded as an indirect marker of malnutrition, it was not associated with an increased risk of recurrence in our analysis.

To our knowledge, this is the first study to assess body composition in cases of recurrent incisional hernia (IH) following surgical repair in liver transplant recipients. As a preliminary observation, our findings suggest that all IHs—including recurrences—should ideally be managed with mesh placement, in accordance with established recommendations for the general population. Nevertheless, the use of a foreign body, such as a prosthetic mesh, in immunocompromised patients raises concerns regarding the risk of infection. Historically, this has contributed to hesitancy surrounding mesh implantation in transplant recipients. However, evidence from the literature does not indicate an increased risk of complications —particularly infections—in this population especially when the repair is performed laparoscopically [26].

In this study, body composition—including skeletal muscle mass index (SMI), subcutaneous fat area (SFA), and visceral fat area (VFA)—was evaluated using an automated method based on artificial intelligence (deep learning) applied to computed tomography (CT) scan data. This approach has demonstrated greater accuracy than body mass index (BMI) in assessing undernutrition [27]. It is well established that muscle mass plays a critical role in the success of liver transplantation [28]. To our knowledge, this is the first study to investigate the relationship between visceral and subcutaneous fat mass and the recurrence of incisional hernia (IH) using this advanced methodology.

Sarcopenia is highly prevalent among patients with advanced chronic liver disease awaiting transplantation, with an estimated prevalence of approximately 40% [18]. It has been associated with an increased risk of postoperative complications and mortality [29]. However, there is currently no universally accepted definition of sarcopenia based on the skeletal muscle mass index (SMI), resulting in substantial heterogeneity across existing studies, as underscored in the meta-analysis by *van Vugt et al.* [30]. Contrary to expectations, our analysis did not reveal a significant association between the presence of sarcopenia and the recurrence of incisional hernia (IH).

Incisional hernia (IH) occurred in nearly one-third of liver transplant recipients—a significantly higher rate than that observed in the non-transplanted population [31]. However, this comparison is limited by differences in follow-up protocols and surgical techniques between the two groups. Moreover, the elevated recurrence rate observed in our study may, in part, be attributable to the higher rate of hernia repair in this population. While *Bosanquet et al.* report a repair rate of only 5.6% in the general population [1], our cohort exhibited a rate of 50%. A higher rate of surgical intervention inherently increases the likelihood of recurrence. The recurrence rate in our study (36.5%) exceeds that reported in the existing literature [25, 32, 33]. This elevated rate may be explained, in part, by the duration of follow-up. According to *Smith et al.*, the risk of developing an incisional hernia can persist for up to 12 years following liver transplantation [34]. In our cohort, the median follow-up period was 6.5 years. This follow-up was conducted alternately by the hepatology and surgical teams, which likely contributed to more accurate detection of hernias. In our view, this multidisciplinary surveillance minimizes the risk of diagnostic bias.

The method of fascial closure may also influence the incidence of incisional hernia (IH). At our center, closure is performed using a continuous suture technique: a median suture is applied to the vertical portion, while a three-layer closure is employed for the transverse segment. *Deerenberg et al.* have demonstrated that the small-bite suture technique significantly reduces the risk of IH [35]. This approach is implemented in our practice, although the suture length-to-wound length ratio is not routinely calculated. Consistent with findings in the literature, primary fascial repair using simple continuous suture at the time of initial IH has been identified as an independent risk factor for recurrence [22, 36]. More recently, *Frountas et al.* reported a 50% reduction in recurrence rates following laparoscopic repair in liver transplant recipients [37].

Immunosuppression is frequently identified as a risk factor for incisional hernia (IH) following organ transplantation [38, 39]. In the context of liver transplantation, mycophenolate mofetil (MMF) and sirolimus are the two primary agents associated with an increased risk of IH [9, 40]. However, our study did not confirm a significant association between these immunosuppressive agents and hernia recurrence. The use of low-dose corticosteroids, their early discontinuation, and the individualized adjustment of MMF based on area under the curve (AUC) monitoring may account for the absence of a statistically significant correlation in our cohort.

According to *Smith et al.*, overweight status (defined as a BMI > 25 kg/m²) is an independent risk factor for incisional hernia (IH), likely due to the increased intra-abdominal pressure it exerts, resulting in greater mechanical stress on the abdominal wall [34]. Our findings are consistent with the literature, as BMI was also associated with a heightened risk of recurrence. However, BMI remains a limited and imprecise indicator of body composition and fat mass distribution [4]. For this reason, it was excluded from our multivariate analysis in favor of more accurate assessments of parietal and visceral adiposity.

Visceral obesity is a well-established risk factor for complications in abdominal surgery [41], yet it remains underrecognized in the context of liver transplantation. Visceral fat area (VFA) is a valuable parameter for assessing this risk; however, standardization of the computed tomography (CT) slice level is essential to ensure reliable comparisons across studies [42, 43]. In the present study, VFA was measured at the level of the third lumbar vertebra (L3), consistent with standard practices for the assessment of sarcopenia. It is worth noting that in this study, visceral obesity appeared to be a potential risk factor for incisional hernia, as the association did not reach the conventional threshold for statistical significance in the Fine and Gray model (*P* = 0.055). This attenuation may be attributed to the limited number of events and the incorporation of the competing risk of death, which reduces the statistical power of the analysis. Although the association did not reach statistical significance, the consistency of the effect size and the direction of the association may suggest a potential clinical relevance of visceral obesity as a risk factor for incisional hernia recurrence. This warrants further investigation in larger cohorts.

Standardization of Hounsfield unit (HU) thresholds for the assessment of visceral fat is also essential. In this study, we applied a range of − 190 to − 30 HU, although other investigations have used narrower intervals, such as − 150 to − 50 HU [44]. Likewise, the definition of visceral obesity remains a topic of ongoing debate. We adopted a threshold of 100 cm²/m², as recommended by the Japan Society for the Study of Obesity [45]. However, the notable morphological differences between Asian and European populations suggest that this threshold may require adjustment to allow for meaningful comparisons across studies.

This study has several limitations. As a retrospective analysis, it is subject to the methodological biases inherent in this study design. Despite rigorous follow-up, the diagnosis of incisional hernia (IH) may have been either overestimated or underestimated, particularly in patients with a lax abdominal wall or significant adipose panniculus—features commonly associated with metabolic syndrome. Moreover, in the absence of clinical complications, computed tomography (CT) scans were not systematically performed, potentially resulting in the underdiagnosis of small or asymptomatic hernias. However, such cases were not clinically relevant and did not warrant intervention [46].

## Conclusion

This study is the first to investigate body composition as a potential risk factor for incisional hernia (IH) recurrence in liver transplant recipients. Although sarcopenia was initially hypothesized to contribute to recurrence, our analysis did not reveal a statistically significant association. This suggests that sarcopenia may not be a major determinant of recurrence risk in this population. In contrast, our findings highlight the potential impact of visceral obesity, which was more consistently associated with recurrence. Given the increasing prevalence of overweight and obesity, targeted pre-transplant interventions addressing visceral adiposity may help reduce the long-term risk of IH recurrence.

## Electronic supplementary material

Below is the link to the electronic supplementary material.

Supplementary Material 1
