# Supplementary material for: Incidence and risk factors for recurrence of incisional hernia repair after liver transplantation: a retrospective cohort study
Source: Hernia. 2025 Jun 27;29(1):217. doi: 10.1007/s10029-025-03400-0 (PMC12204869; doi:10.1007/s10029-025-03400-0)
Supplement: Supplementary file 1 — Supplementary Material 1 [file 10029_2025_3400_MOESM1_ESM.docx]

**Figure S1 Actuarial analysis of the occurrence of incisional hernia and recurrence according Kaplan-Meier**

**A**

**B**


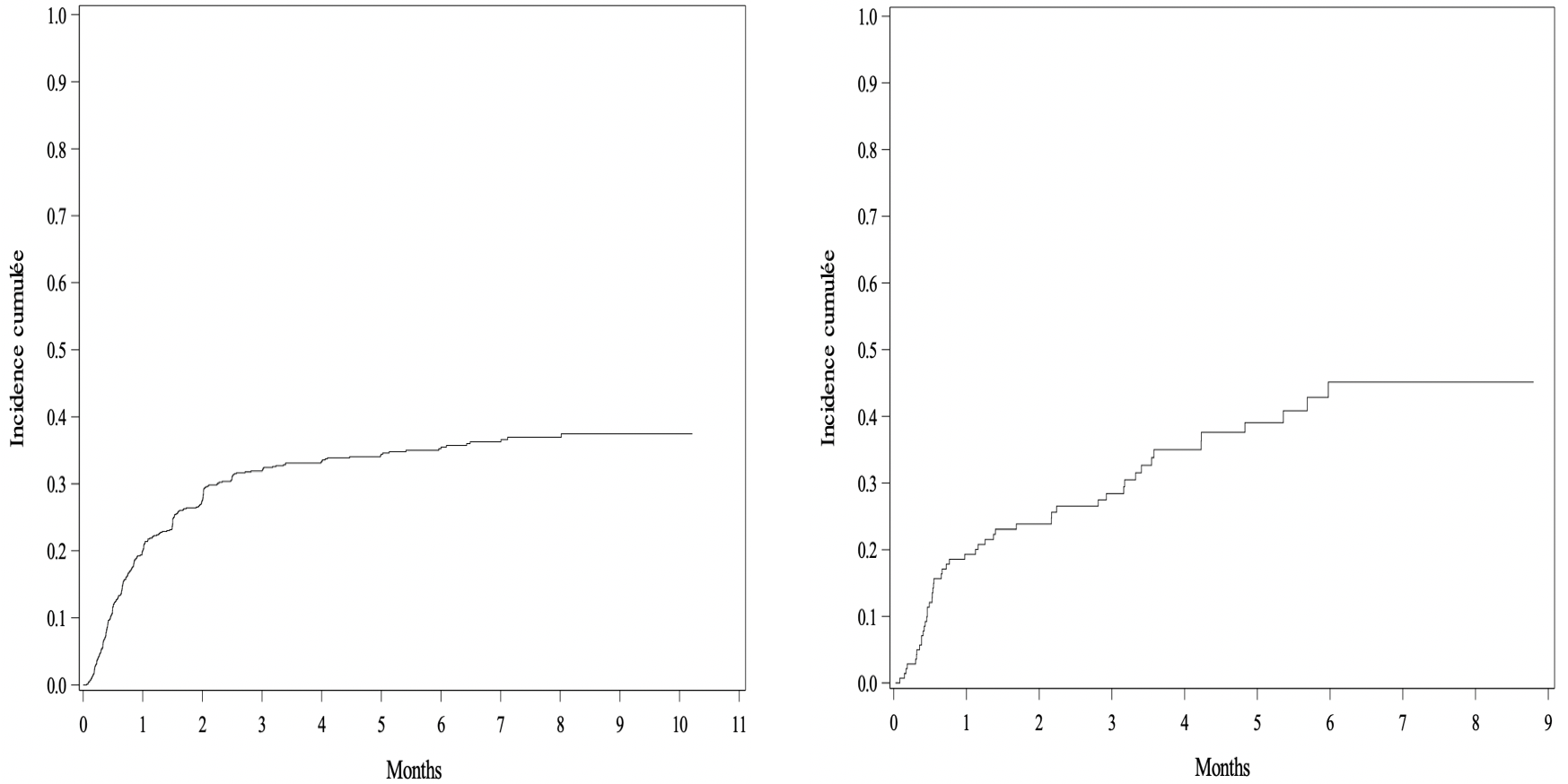


***A :*** *Cumulative incidence of IH.* ***B :*** *Cumulative incidence of recurrence.*

A B

***A :*** Cumulative incidence of incisional hernias. (with the event of interest being the incisional hernia, and J0 being the date of liver transplantation). ***B***Cumulative incidence of recurrences (with the event of interest being the recurrence, and J0 being the date of repair of the first incisional hernia).
